# Association between *TBXT* rs2305089 polymorphism and chordoma in Iranian patients identified by a developed T‐ARMS‐PCR assay

**DOI:** 10.1002/jcla.24150

**Published:** 2021-11-27

**Authors:** Maryam Jalessi, Mohammad Saeed Gholami, Ehsan Razmara, Sajad Hassanzadeh, Alireza Sadeghipour, Amin Jahanbakhshi, Alireza Tabibkhooei, Eshagh Bahrami, Masoumeh Falah

**Affiliations:** ^1^ Skull Base Research Center The Five Senses Health Institute Hazrat Rasoul Akram Hospital Iran University of Medical Sciences Tehran Iran; ^2^ ENT and Head and Neck Research Center and Department The Five Senses Health Institute Hazrat Rasoul Akram Hospital Iran University of Medical Sciences Tehran Iran; ^3^ Department of Hematology and Blood Transfusion School of Paramedical Sciences Shiraz University of Medical Sciences Shiraz Iran; ^4^ Australian Regenerative Medicine Institute Monash University Clayton Victoria Australia; ^5^ Pathology Department Rasoul Akram Hospital Iran University of Medical Sciences Tehran Iran; ^6^ Department of Neurosurgery Rasoul Akram Hospital Iran University of Medical Sciences Tehran Iran

**Keywords:** chordoma, rs2305089, SNP, T‐ARMS‐PCR, TBXT

## Abstract

**Background:**

Chordoma is a locally aggressive bone tumor with a high capability of recurrence. Because chordoma often occurs at critical locations next to neurovascular structures, there is an urgent need to introduce validated biomarkers. *T*‐*box transcription factor T* (*TBXT*; OMIM: 601397) plays an important role in the pathogenesis and survival of chordoma cells.

**Methods:**

Herein, we aimed to show whether rs2305089 polymorphism is correlated with chordoma in the Iranian population. In order to detect rs2305089, tetra‐primer amplification refractory mutation system‐polymerase chain reaction (T‐ARMS‐PCR) was used. In total, 19 chordoma patients and 108 normal healthy individuals were recruited and screened using T‐ARMS‐PCR. The results were subsequently validated by Sanger sequencing.

**Results:**

The genotype distributions and allele frequencies were significantly different among the patient and healthy groups (*p*‐value <0.05). The A allele of rs2305089 showed a significant positive association with chordoma risk (*p*‐value <0.05). DNA sequencing verified the T‐ARMS‐PCR results as well. This study demonstrated the association between *TBXT* rs2305089 and chordoma in an Iranian population using a simple, accurate, and cost‐effective T‐ARMS‐PCR assay.

**Conclusions:**

Our results were in line with those of previous studies showing that *TBXT* rs2305089 is associated with chordoma development. We also developed an efficient T‐ARMS‐PCR assay to determine the genotype of rs2305089.

## Graphical abstract

This study aimed to show whether rs2305089 polymorphism is associated with chordoma in the Iranian population. In order to detect the rs2305089, tetra‐primer amplification refractory mutation system‐polymerase chain reaction (T‐ARMS‐PCR) was used. In total, 19 chordoma patients and 108 normal healthy individuals were recruited and screened using T‐ARMS‐PCR. Besides, the results were validated by Sanger sequencing. This study demonstrated the association of *TBXT* rs2305089 with chordoma in an Iranian population using a simple, accurate, and cost‐effective T‐ARMS‐PCR assay. Our results were in line with the previous studies showing that *TBXT* rs2305089 is associated with chordoma development. We also developed an efficient T‐ARMS‐PCR assay so as to determine the genotype of rs2305089.

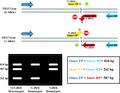

## 1 INTRODUCTION

Chordoma (OMIM: 215400) is a rare primary bone tumor that originated from notochord remnants—a rod‐like structure that supports embryo development—in the axial skeleton.^1^, ^2^, ^3^ Chordoma usually progresses slowly, but its locally aggressive nature can affect the patients’ quality of life.^4^ This kind of cancer often occurs sporadically, but some rare familial cases have also been documented.^5^ The estimated overall incidence of chordoma in the general population is 0.08 per 100,000 live births.^4^ This cancer affects males more frequently than females (2:1).^6^ Although chordoma's occurrence has been reported from infancy to senescence, it often occurs in the sixth decade of patients’ life.^2^ Furthermore, its occurrence tends to be higher in Caucasians than in African‐Americans (4:1).^2^

The first‐line strategies to treat primary chordoma are still based on surgical resection followed by radiotherapy, although these strategies result in a median overall survival of 7.7 years.^7^, ^8^ Moreover, chordoma, as a mildly aggressive cancer, often affects the adjacent vital tissues before becoming symptomatic—thus, completely resecting all cancer‐related tissues is demanding. In short, there is an urgent need to introduce safe biomarkers (e.g., susceptible genetic loci) to better manage patients as accurately as possible.

*TBXT* (*T*‐*box transcription factor T*; OMIM: 601397) has been introduced as one of the most important genes in embryonic development. This gene is exclusively expressed during the early stages of development and is silenced in most developed tissues, except differentiated tissues of the testis and some parts of the thyroid.^9^, ^10^ *TBXT*—also known as the T gene or brachyury—functions as an embryonic nuclear transcription factor in mesoderm formation and differentiation. This protein binds to the palindromic T‐site of DNA using its N‐terminus (T‐box).^11^ Interestingly, *Tbxt* ^−/−^ mice die during the embryonic period due to multiple mesodermal abnormalities.^11^

The *TBXT* is located on the 6q27 region and is associated with susceptibility to chordoma and neural tube defects.^5^, ^9^, ^12^, ^13^ For example, the homozygous H171R was observed in four members of three unrelated consanguineous families with sacral agenesis and vertebral anomalies.^14^ Germline duplication of *TBXT* was also observed in the familial type of chordoma.^5^ Moreover, the silencing of TBXT in chordoma cell line U‐CH1 resulted in decreased cell proliferation.^12^

Pillay et al. indicated a strong association between chordoma risk and the common nonsynonymous single‐nucleotide polymorphism (SNP) rs2305089 in Europeans.^1^ Rs2305089 (NM_003181.4; c.530G>A: p. Gly177Asp) is located at exon 4 of *TBXT*. The researchers also indicated a higher mRNA expression of *TBXT* in patients with AA genotype than in GA sporadic chordoma patients without *TBXT* gene duplication.^1^ The higher susceptibility imputed to the A allele to chordoma was later confirmed by Kelley et al. in American and Canadian individuals.^3^ On the contrary, a study using 65 skull‐based chordoma patients and 120 healthy individuals among the Chinese population showed that rs2305089 was not significantly correlated with chordoma risk.^15^ Since the reported association of rs2305089 may be linked to ‘population‐specific’ backgrounds, it is imperative to check this association in different ethnic populations.

Hence, finding disorder‐associated alleles/genotypes is important to reduce the burden of genetic disorders and take steps toward turning ‘generalized’ medicine into ‘personalized’ medicine. Although using high‐throughput approaches (e.g., genome‐wide association studies, TaqMan genotyping assay, and whole‐exome sequencing) is highly suggested for genetic association study, they are not cost‐affordable for limited resources. Such techniques, in some cases, can be replaced by inexpensive, simple, and quick ones, such as tetra‐primer amplification refractory mutation system‐polymerase chain reaction (T‐ARMS‐PCR). T‐ARMS‐PCR amplifies the specific target template by only two sets of primers, including inner and outer ones. The outer primers are non‐allele‐specific and simply amplify the target sequences of the main template, whereas the inner primers are allele‐specific ones whose 3′‐ends embrace the polymorphic nucleotides.

The present study investigated the association between *TBXT* rs2305089 polymorphism and chordoma in an Iranian population. Herein, we also aimed to develop a reliable, low‐cost, and simple T‐ARMS‐PCR method to ease the genotyping of this polymorphism.

## 2 MATERIALS AND METHODS

### 2.1 Study participants

In order to determine whether rs2305089 is associated with chordoma susceptibility, this study assessed a total of 127 Iranian individuals (19 chordoma patients and 108 healthy controls). The study protocol was approved by the ethics committee of Iran University of Medical Sciences, Tehran, Iran, under the ethics code of “IR.IUMS.REC.1399–901.” All participants provided written informed consent after the study protocol had been completely explained. The study is in line with the Declaration of Helsinki Ethical Principles. Chordoma was diagnosed based on patients’ clinical history, computed tomography scans, magnetic resonance imaging with gadolinium‐based contrast agents, and immunohistochemistry. The control individuals had no history of chordoma or any other cancers.

### 2.2 DNA Extraction

A blood sample of approximately 10 ml was collected from each participant, and their DNA samples were isolated using a standard salting‐out procedure.^16^, ^17^ The quality and quantity of the DNA samples were determined with a 1.5% agarose gel and NanoDrop 2000c spectrophotometer (Thermo Fisher Scientific, Clayton, VIC, Australia), respectively.

### 2.3 Primer designing and T‐ARMS‐PCR

The DNA sequence of the *TBXT* gene was retrieved from the NCBI database according to Human Genome Reference hg19 (NM_003181.4). Subsequently, primers were designed for selected SNP using Primer1 software (Table 1).^18^ The specificity of each primer was assessed using ‘NCBI Primer BLAST’ (https://www.ncbi.nlm.nih.gov/tools/primer‐blast/ ) and *in*‐*silico* PCR of UCSC genome browser (https://genome.ucsc.edu/cgi‐bin/hgPcr).

**TABLE 1:** Primer sequences and product size of the T‐ARMS‐PCR assay for rs2305089

Gene	Polymorphism	Primer sequence (5′–3′)	Product size
*TBXT*	rs2305089	F‐outer−5́‐CCGTTGTCTAGCCCTAAACTC−3́	Control 810 bp
		R‐outer−5́‐GTTCTCTCCTGTGCTTCCATTT−3́	
		F‐inner−5́‐CACATAGTGAGAGTTGGTGG−3́	G allele 262 bp
		R‐inner−5́‐GGTGATCATGCGCTGTGTAT−3́	A allele 587 bp

T‐ARMS‐PCR was performed in a total volume of 20 μl, including 10 μl of 2× Taq DNA Polymerase Master Mix RED (Ampliqon, Odense, Denmark), 0.8 μM of each outer primer, 1.5 μM of each inner primer, 1 μl of DNA template, and 4.4 μl of ddH2O. PCR was performed with the initial denaturation at 95°C for 5.0 min, followed by 32 cycles at 95°C for 45 sec, at 56°C for 30 sec, at 72°C for 45 sec, and a final extension at 72°C for 5.0 min. The PCR products were visualized by ethidium bromide staining and 1.5% agarose gel electrophoresis.

### 2.4 Sanger sequencing

Sanger sequencing was used to validate the T‐ASMS‐PCR results. To this end, 15 samples showing the GG, GA, and AA genotype by T‐ARMS‐PCR were amplified and sequenced. To amplify the template, we used the same outer primer sets as mentioned above.

### 2.5 Statistical Analysis

The statistical analysis was performed using SPSS software version 24.0 (SPSS Inc.,). A chi‐square (χ^2^) test was used to compare the allele and genotype frequencies between patients and control groups. Odds ratios (OR) and 95% confidence intervals (CI) were calculated using χ^2^ test. *p*‐values <0.05 were considered statistically significant.

## 3 RESULTS

### 3.1 Demographic data

The median (IQR) ages of patients and control participants were 52.0 (16–80) years and 47.0 (30–83) years, respectively. The patient group comprised of six females and 13 males, while the control group consisted of 55 females and 53 males. The patient‐gender distribution was compatible with those reported in previous reports.^6^ We could not find any statistically significant differences between the two groups in terms of their age (*p*‐value =0.4) and gender (*p*‐value =0.1). The most important clinical findings detected in the patients are put forth in Table 2.

**TABLE 2:** Summary of clinical characteristics of chordoma patients

Case Number	Age	Sex	Location	Subtype	Surgical approach	Disease status
**1**	80	M	Skull base	Conventional	EEA	Recurrence
**2**	48	F	Skull base‐Sacrum	Conventional	Posterior	Recurrence
**3**	77	F	Mobile spine	Conventional	Anterior	Recurrence
**4**	56	F	Sacrum	Conventional	Posterior	Recurrence
**5**	62	M	Skull base	Conventional	EEA	Recurrence
**6**	53	F	Skull base	Conventional	EEA	Primary
**7**	26	M	Skull base	Conventional	Retrosigmoidal	Recurrence
**8**	54	M	Skull base	Conventional	EEA	Primary
**9**	40	M	Sacrum	Conventional	Posterior	Recurrence
**10**	50	F	Skull base	Conventional /Chondroid	Retrosigmoidal	Recurrence
**11**	69	F	Sacrum	Conventional	Posterior	Recurrence
**12**	52	M	Skull base	Conventional	Transoral	Primary
**13**	66	M	Sacrum	Conventional	Posterior	Primary
**14**	43	M	Skull base	Conventional /Chondroid	EEA	Primary
**15**	70	M	Skull base	Conventional	EEA	Primary
**16**	48	M	Sacrum	Conventional	Posterior	Recurrence
**17**	16	M	Skull base	Conventional	Posterior	Primary
**18**	21	M	Skull base	Conventional /Chondroid	EEA	Primary
**19**	45	M	Skull base	Conventional	EEA	Recurrence

Abbreviations: EEA, Endoscopic endonasal approach; M, Male; F, Female.

### 3.2 T‐ARMS‐PCR

**FIGURE 1:**
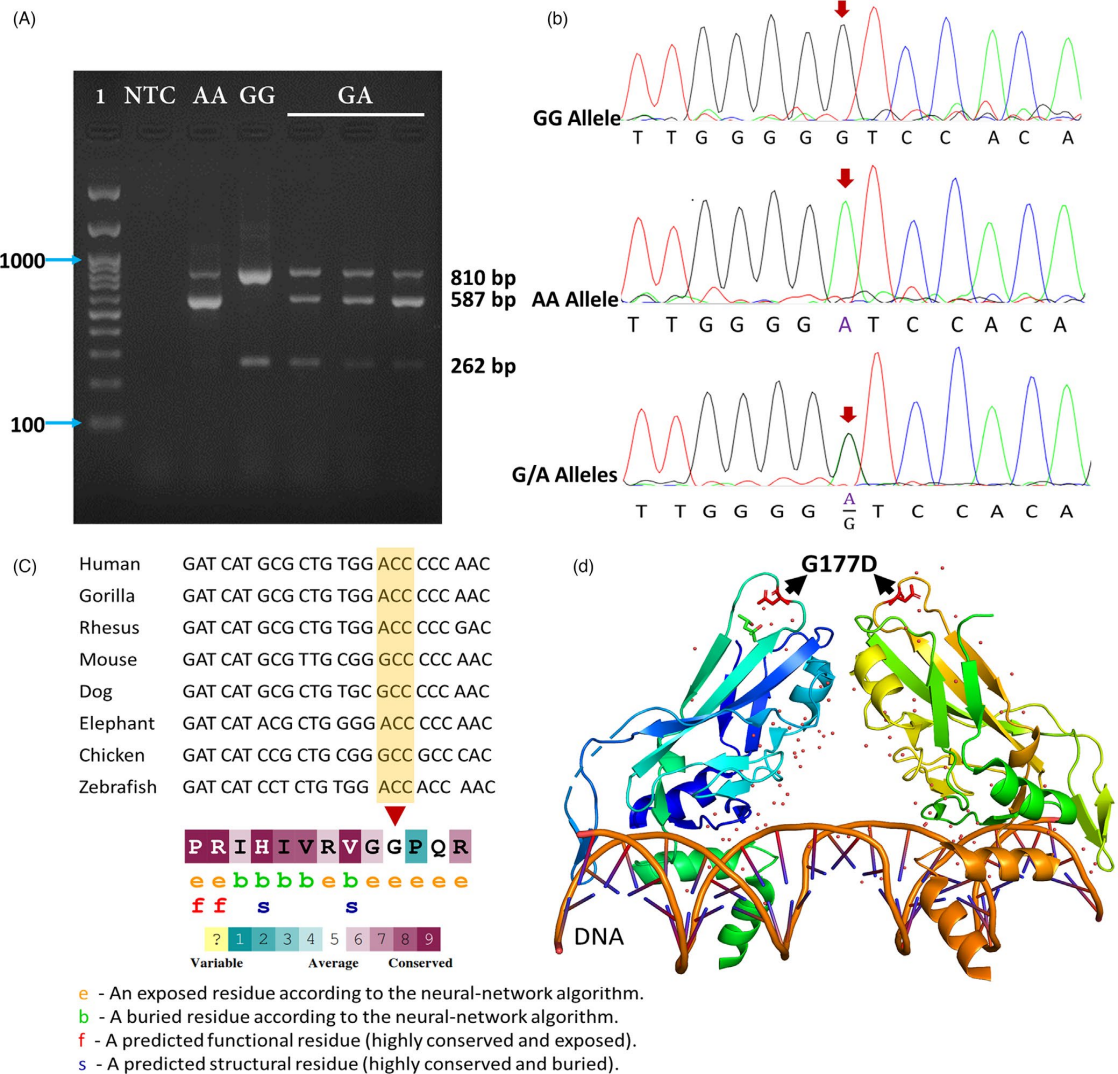
The results of rs2305089 genotyping. (A) Electrophoretogram of the T‐ARMS‐PCR products of rs2305089. The GA genotype (810 bp, 587 bp, and 262 bp) is shown in lanes 5–7, respectively. The GG genotype (810 bp and 262 bp) is shown in lane 4, and the AA genotype (810 bp and 587 bp) is indicated in lane 3. In this figure, 1: ladder, NTC: No Template Control. (B) Sanger sequencing results of GG, AA, and GA genotype of rs2305089. (C) Evolutionary conservation is indicated using a UCSC multiple sequences alignment. (D) Three‐dimensional structure of TBXT protein shows the position of G177D polymorphism. We used the PDB structure under the code “6F59”

A T‐ARMS‐PCR electrophoretogram of rs2305089 is shown in Figure 1A. The GA genotype showed three bands (262 bp, 587 bp, and 810 bp), while the GG genotype showed two bands (262 bp and 810 bp). Also, the AA genotype showed two bands (587 bp and 810 bp).

The genotype distributions and allele frequencies of rs2305089 in patients and control participants are summarized in Table 3. Genotype distributions of participants were in line with the Hardy–Weinberg equilibrium (χ^2^ = 0.063; *p*‐value =0.96).

**TABLE 3:** Genotype, allele frequencies, and recessive model of rs2305089 in the study population

	Case% (*N* = 19)	Control% (*N* = 108)	χ^2^	*p*‐value	OR (95% CI)
Genotype					
GG	2 (10.5%)	26 (24.1%)	8.4	0.015	
GA	7 (36.8%)	59 (54.6%)			
AA	10 (52.6%)	23 (21.3%)			
Recessive model					
AA	10 (52.6%)	23 (21.3%)	8.2	0.004	4.1 (1.5–11)
GG+GA	9 (47.4%)	85 (78.7%)			
Allele					
G	11 (28.9%)	111 (51.4%)	6.5	0.011	2.59 (1.2–5.4)
A	27 (71.1%)	105 (48.6%)			

The allele and genotype frequencies of the rs2305089 were significantly different between the patient and control participants. Specifically, the rs2305089 AA genotype (OR = 4.1, 95% CI: 1.5–11, *p*‐value =0.004) had a significant association with chordoma, and the A allele of rs2305089 was significantly associated with a higher risk of chordoma in the patients (OR = 2.59, 95% CI: 1.2–5.4, *p*‐value =0.011) (Table 3).

### 3.3 DNA sequence analysis verified the results of the T‐ARMS‐PCR assay

To verify the results obtained by the T‐ARMS‐PCR assay, we used Sanger sequencing for genotyping 15 representative samples (Figure 1B). The findings of the DNA sequencing were completely consistent with the results of T‐ARMS‐PCR (Figure 1). To show the heterogeneity of our targeted rs2305089, DNA sequences of only 3 samples are shown in Figure 1.

## 4 DISCUSSION

*TBXT* gene encodes an embryonic nuclear transcription factor that is necessary for proper mesoderm formation and differentiation. As an evolutionarily conserved gene (Figure 1C,1D), *TBXT* controls the development of the notochord and is then silenced during later developmental stages (e.g., in the human fetus at ~12 weeks). Therefore, the notochord recedes prenatally.^9^, ^19^

*TBXT* variations have been associated with higher susceptibility to chordoma and neural tube defects.^5^, ^14^ Although *TBXT* expression is aberrantly changed in other tumors, its expression is a diagnostic hallmark of chordoma.^9^, ^20^, ^21^ The upregulation of *TBXT* may result in increased expression of mesenchymal markers, decreased expression of epithelial markers, and promoted cell migration and invasion. Furthermore, TBXT can promote the epithelial–mesenchymal transition.^7^ Also, to some extent, it controls the cell cycle and biological behavior of cancer cells.^22^

Herein, we unveiled a significant association between chordoma and the presence of the AA genotype of rs2305089 in the *TBXT* (OR = 4.1, 95% CI: 1.5–11, *p*‐value =0.004). This finding is in line with the findings of Pillay et al. on European ancestors and similar investigations on American or Canadian populations.^1^, ^3^ In a study conducted on the Chinese skull‐based chordoma patients, rs2305089 was not significantly correlated with increased risk of chordoma, suggesting that circumstances can be attributed to the cancer site (i.e., strictly skull‐based chordoma) and ethnicity.^15^ Conversely, Kelley et al. used a majority of cases of skull‐based chordoma and showed a significant association between chordoma and rs2305089.^3^

In the present study, we indicated that the A allele contributes to a higher risk of chordoma development. The frequency of the A allele of rs2305089 was estimated at 71% among our chordoma patients and 49% in the control group (OR = 2.59, 95% CI: 1.2–5.4, *p*‐value = 0.011). The frequency of the risk allele (A) among controls was similar to the frequencies presented in previous studies (~53%).^1^, ^3^ The A allele frequency in the chordoma group (71%) was consistent with the findings of Kelley et al. (72% among familial cases and 76% in sporadic cases). However, A allele frequency was higher among chordoma patients in the report of Pillay et al. (87.5% in the discovery set and 82.5% in the replication set). In fact, the association for rs2305089 cannot be affected by the age of patients and the location of the tumor,^3^ meaning that such differences in allele frequency can be attributed to the population/sample size. Moreover, the A allele frequency in South Asia is more similar to that of Caucasians (~50%) than among East Asian population (36%).^23^ This might explain why the Chinese population showed no association with rs2305089, which conflicts with other reports.

SNP genotyping and genotypes’ associations with increased risk of disease, prognosis, and influenced responses to the treatment have been investigated carefully in cancer studies.^1^, ^7^, ^24^, ^25^ Also, emerging high‐throughput techniques that are compatible with a large sample size make SNP genotyping ideal for biomarker findings. On the contrary, due to the high cost of such techniques, their applications are limited.

**FIGURE 2:**
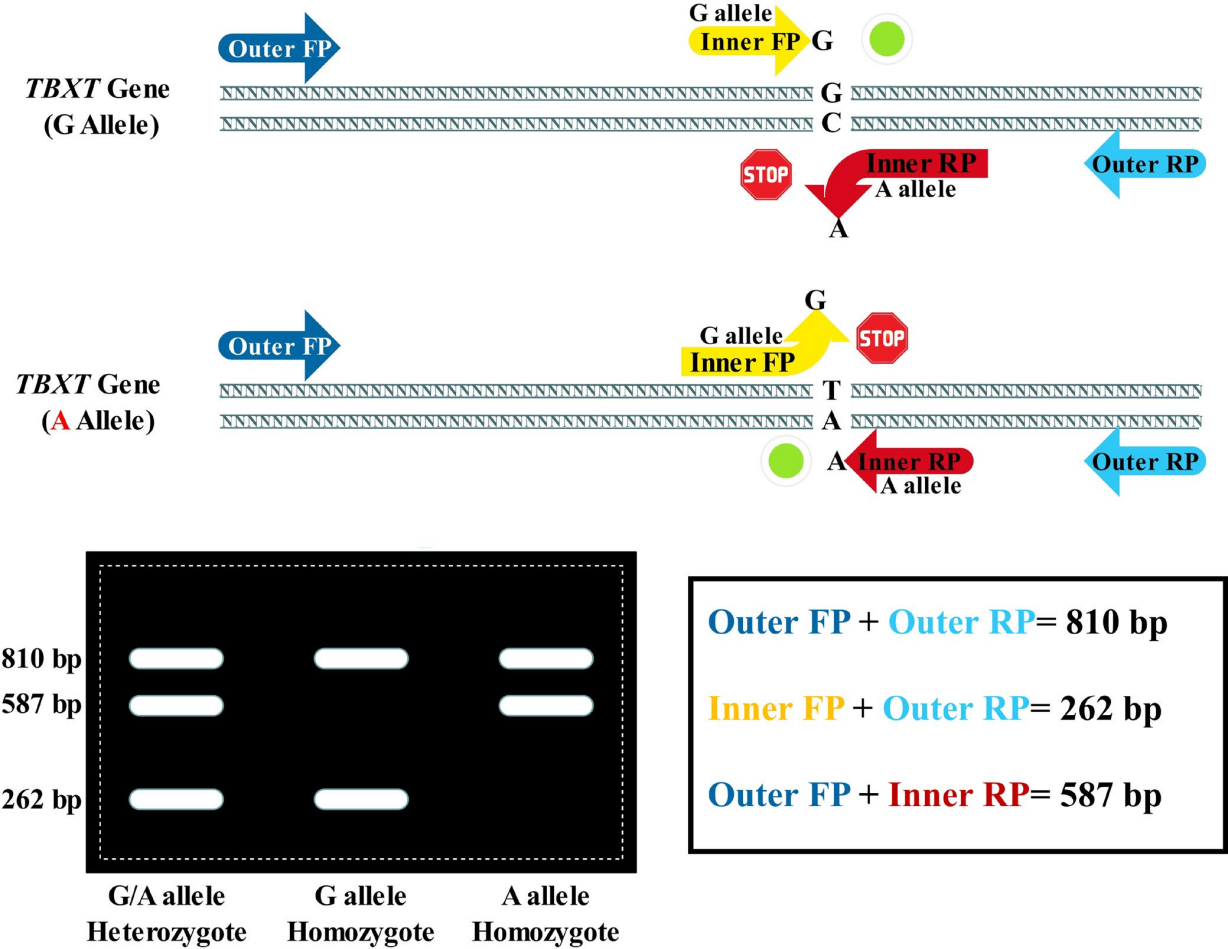
Schematic depiction of the tetra‐primer ARMS‐PCR assay for rs2305089 genotyping. An illustration shows how the rs2305089 can be detected using T‐ARMS‐PCR and also demonstrates the genotyping patterns for each genotype

In the present study, we developed a T‐ARMS‐PCR for genotyping rs2305089 in chordoma patients, which was confirmed using a gold standard technique, Sanger sequencing. Different techniques based on conventional PCR for SNP genotyping have been developed, among which T‐ARMS‐PCR is distinguished.^26^ Using T‐ARMS‐PCR is advantageous because it can be run easily, quickly, and cheaply. Indeed, T‐ARMS‐PCR is more affordable than other similar techniques that have been developed to detect SNPs, such as polymerase chain reaction‐restriction fragment length polymorphism (PCR‐RFLP), single‐strand conformation polymorphism (SSCP), and allele‐specific PCR (ASPCR). Moreover, unlike DNA direct sequencing, pyrosequencing, TaqMan assay, and high‐resolution melting analysis, T‐ARMS‐PCR does not need special expensive tools.^27^ To the best of our knowledge, the present study is the first to use T‐ARMS‐PCR to determine the rs2305089 genotype (Figure 2).

The A allele is located within conserved DNA‐binding domain of transcription factor TBXT^28^; this domain is in the N‐terminal region between the residues of 42–219. Cheng et al. used support vector machines to indicate that the rs2305089 can decrease the stability of the protein.^29^ Pillay et al. showed that the functions of TBXT can be changed due to this variant (i.e., the AA genotype is correlated with different expression levels of *TBXT* and its downstream targets).^1^ In other words, a higher mRNA level of TBXT via the AA genotype was imputed to the expression of downstream targets in chordoma.^1^

There is no approved therapeutic strategy for chordoma, and current treatments are often based on surgery, followed by radiotherapy.^3^, ^30^ *TBXT* has been reported as a definitive diagnostic biomarker, as chordoma cells need TBXT to survive and maintain their invasive phenotype.^12^, ^31^ Moreover, due to the low *TBXT* expression in most normal adult tissues, exclusive cancer‐specific expression, and its roles in epithelial–mesenchymal transition, *TBXT* has been considered as a therapeutic target for chordoma. For example, different molecular target therapies have been performed, for example, it has been identified that using small molecules that target *TBXT* ^32^ or doing immunotherapy with *TBXT* vaccine^33^ can be beneficial to treat chordoma. Additionally, some studies have used short hairpin RNAs to knock down *TBXT*,^12^, ^31^ while others target several components in relation to *TBXT*.^34^, ^35^ In addition, it may have some effects on survival rate in spinal column chordoma patients, as Bettegowda et al. indicated significantly improved overall survival in chordoma patients with the A variant at rs2305089.^7^

One of the limitations of our study is the limited number of patients that in turn can be attributed to this fact that chordoma is a rare disease. Our data were fully consistent with the previous findings, showing that this study has adequate power to arrive at a conclusion. Another limitation is that we did not determine the effects of rs2305089 on the expression of *TBXT* or other downstream genes. Further investigations should be conducted to determine the RNA and protein levels of TBXT in the patients.

## 5 CONCLUSIONS

T‐ARMS‐PCR is a sensitive, specific, and cost‐effective technique for SNP genotyping. We indicated a significant association of rs2305089 with chordoma development in Iranian patients by developing the T‐ARMS‐PCR method. The validity of this method was 100% concordant with DNA sequencing. As TBXT is becoming an attractive target for molecular targeted therapy in chordoma, a simple and reliable T‐ARMS‐PCR offers an effective screening method for rs2305089 in these patients and might pave the way for new molecular targeted therapy techniques. It could also improve diagnosis management and counseling provided to patients with chordoma.

## CONFLICT OF INTEREST

The authors declare no conflict of interest.

## Data Availability

Data sharing not applicable to this article as no datasets were generated or analyzed during the current study.
